# Physiological and therapeutic regulation of glucose homeostasis by upper small intestinal PepT1-mediated protein sensing

**DOI:** 10.1038/s41467-018-03490-8

**Published:** 2018-03-16

**Authors:** Helen J. Dranse, T. M. Zaved Waise, Sophie C. Hamr, Paige V. Bauer, Mona A. Abraham, Brittany A. Rasmussen, Tony K. T. Lam

**Affiliations:** 1 Toronto General Hospital Research Institute, UHN, Toronto, ON M5G 1L7 Canada; 2 Department of Physiology, University of Toronto, Toronto, ON M5S 1A8 Canada; 3 Department of Medicine, University of Toronto, Toronto, ON M5S 1A8 Canada; 4 Banting and Best Diabetes Centre, University of Toronto, Toronto, ON M5G 2C4 Canada

## Abstract

High protein feeding improves glucose homeostasis in rodents and humans with diabetes, but the mechanisms that underlie this improvement remain elusive. Here we show that acute administration of casein hydrolysate directly into the upper small intestine increases glucose tolerance and inhibits glucose production in rats, independently of changes in plasma amino acids, insulin levels, and food intake. Inhibition of upper small intestinal peptide transporter 1 (PepT1), the primary oligopeptide transporter in the small intestine, reverses the preabsorptive ability of upper small intestinal casein infusion to increase glucose tolerance and suppress glucose production. The glucoregulatory role of PepT1 in the upper small intestine of healthy rats is further demonstrated by glucose homeostasis disruption following high protein feeding when PepT1 is inhibited. PepT1-mediated protein-sensing mechanisms also improve glucose homeostasis in models of early-onset insulin resistance and obesity. We demonstrate that preabsorptive upper small intestinal protein-sensing mechanisms mediated by PepT1 have beneficial effects on whole-body glucose homeostasis.

## Abstract (web summary)

High protein diets are known to improve metabolic parameters including adiposity and glucose homeostasis. Here the authors demonstrate that preabsorptive upper small intestinal protein-sensing mechanisms mediated by peptide transporter 1 improve glucose homeostasis by inhibiting hepatic glucose production.

## Introduction

The global prevalence of diabetes is increasing at an alarming rate, with >400 million people currently affected by diabetes, and it is estimated that by 2030 diabetes will be the seventh leading cause of death^1^. Type 2 diabetes is a heterogeneous disease that results from both environmental and genetic factors, which ultimately lead to the dysregulation of energy balance and glucose homeostasis. Hyperglycemia, a key characteristic of diabetes, generally results from insulin resistance leading to increased hepatic glucose production (GP) and impaired glucose uptake. As hyperglycemia contributes to the development of diabetic-related complications such as blindness, renal failure, cardiovascular disease, and stroke, there is an increasingly urgent need to identify novel therapeutic strategies to restore glucose homeostasis and/or reduce blood glucose levels in diabetic individuals.

In this regard, exhaustive studies conducted in rodents and humans have demonstrated that high protein (HP) diets improve glucose homeostasis. For example, increased HP intake over several weeks improves metabolic parameters, including body weight, adiposity, insulin sensitivity, glycated hemoglobin levels, and food intake in both humans and rodents^2–6^. While the demonstrated improvements in glucose homeostasis might result secondary to decreased food intake and weight loss, 5 weeks of HP feeding in diabetic patients improved glucose tolerance even when individuals maintained a stable weight^7^ and pair-feeding to match energy intake and body weight improved glucose homeostasis in rats fed a HP diet^8^. This suggests that HP diets regulate glucose homeostasis independent of effects on food intake and/or body composition. Consistent with this, acute short-term HP intake lowers post-prandial glucose levels compared to low protein (LP) intake in both healthy humans^9–11^ and rodents^12–14^. Notably, acute HP feeding is also effective at lowering blood glucose levels and improving glycemic control in individuals with diabetes^7,15–18^. It has been postulated that these improvements in glucose control result from decreased dietary carbohydrate content; however, the addition of protein to a meal reduces the post-prandial glucose response compared to a meal consisting of equal carbohydrate content alone^10,18^ and consumption of a premeal protein beverage reduces post-meal glycemia^19,20^. This suggests that the glucoregulatory influence of acute HP intake results from the presence of protein itself.

Of note, the gastrointestinal tract is the primary site of interaction between incoming nutrients and the body and provides a site for early negative feedback on metabolic homeostasis. Intra-small intestinal amino acid or protein infusion decreases energy intake^21,22^, and it is believed that intestinal protein-sensing mechanisms mediate this effect through a gut–brain axis, whereby gut peptide release activates vagal afferent firing. In line with this, peptone treatment stimulates gut peptide release from intestinal enteroendocrine cells^23–26^, and clinical studies have shown that dietary protein intake or intestinal protein administration increases circulating levels of gut peptides, including cholecystokinin (CCK), glucagon-like peptide-1 (GLP-1), and peptide YY (PYY)^22,27–29^. Furthermore, in vivo intra-small intestinal infusion of protein hydrolysate activates CCK-dependent vagal afferent firing in rodents^30,31^, and HP intake leads to activation of CCK-responsive neurons in the nucleus of the solitary tract^32^. Several sensory receptors in the small intestine might play a potential role in the activation of such a gut–brain axis to regulate food intake. One such candidate is peptide transporter 1 (PepT1), a high-capacity, low-affinity intestinal transporter that is expressed on the apical membrane of enterendocrine cells and that is considered the primary oligopeptide transporter in the small intestine^33^. Notably, PepT1 activation in vitro or in organoid cultures results in gut peptide release^25,34,35^, and inhibition of PepT1 blocks the activation of vagal afferent fibers in response to intra-duodenal protein hydrolysate infusion^30^. However, whether intestinal protein-sensing mechanisms and/or small intestinal PepT1 activation influence glucose homeostasis have not yet been investigated.

**Fig. 1:**
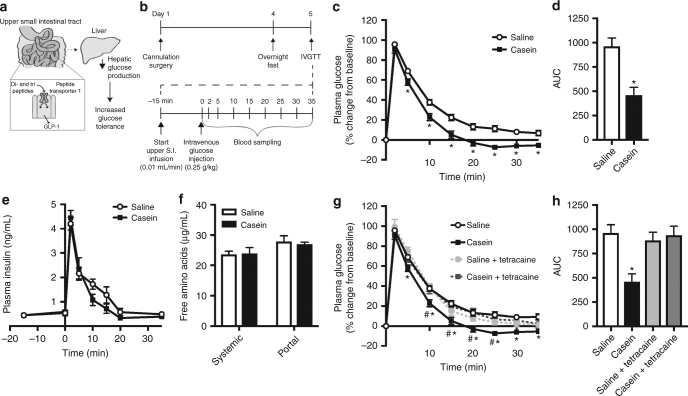
Upper small intestinal infusion of casein hydrolysate increases glucose tolerance via preabsorptive mechanisms in healthy rodents. Schematic of hypothesis (**a**). Glucose tolerance was assessed in conscious, unrestrained healthy rats using an intravenous glucose tolerance test (IVGTT) as outlined in (**b**). Percentage of change in plasma glucose levels (**c**), integrated area under the curve (AUC, **d**), and plasma insulin levels (**e**) over time during the IVGTT in rats that received an upper small intestinal (S.I.) infusion of saline (*n* = 18) or 8% casein hydrolysate (pH 5.0, *n* = 24). Systemic and portal free amino acid levels were assessed in plasma obtained from rats following 50 min of upper S.I. saline (*n* = 6) or casein (*n* = 9) infusion (**f**). Percentage of change in plasma glucose levels (**g**) and integrated AUC (**h**) for rats that received an upper S.I. infusion of saline, casein, saline+tetracaine (*n* = 5) or casein+tetracaine (*n* = 8) during the IVGTT. Values are presented as mean ± s.e.m., where asterisk (*) represents *p* < 0.05 compared to saline control and hash (#) represents p<0.05 compared to casein+tetracaine. Statistical significance was determined using an unpaired, two-tailed *t*-test (two groups) or ANOVA with Tukey post-hoc test (3+ groups)

Collectively, the available evidence suggests that intestinal protein-sensing mechanisms play a role in mediating the effectiveness of HP intake on improving glucose tolerance in both healthy and diabetic rodents and humans (Fig. 1a). Therefore, the objective of this study was to investigate the physiological and therapeutic relevance of intestinal protein-sensing mechanisms on glucose homeostasis in rodents. Herein we demonstrate that acute administration of upper small intestinal protein reduces GP and increases glucose tolerance via PepT1- and GLP-1-mediated protein-sensing mechanisms. Furthermore, we discover both the post-prandial physiological and unique therapeutic relevance of upper small intestinal protein sensing.

## Results

### Upper small intestinal protein sensing regulates glucose homeostasis

We first investigated the potential glucoregulatory role of upper small intestinal protein sensing on whole-body glucose homeostasis under physiological conditions. To do this, we performed an intravenous glucose tolerance test (IVGTT, Fig. 1b) in healthy rodents that received an upper small intestinal infusion of 8% (w/v) casein hydrolysate, a milk-derived peptone and common dietary protein source. Of note, 8% casein hydrolysate was solubilized in distilled water by titration to pH 1.0 and then raised to a pH of 5.0, the native pH of the upper small intestine^36^. As there was no difference in glucose tolerance between rats that received a direct 50 min of intra-upper small intestinal saline or water (pH 5.0) infusion (Supplementary Fig. 1a), saline was used as a control for all future experiments. We observed an increase in glucose tolerance in rats following intra-upper small intestinal casein infusion compared to saline-infused control animals. Importantly, casein infusion resulted in lower blood glucose levels compared to saline as early as 5 min following injection of the glucose bolus, and this was maintained for the duration of the experiment (Fig. 1c). Consistent with this, the integrated area under the curve (AUC) of plasma glucose levels collapsed over time was significantly lower for casein-infused rats (~50% reduction) compared to saline control (Fig. 1d). The improvement in glucose tolerance following casein infusion was independent of differences in post-surgical body weight and plasma glucose and insulin levels, which were comparable between groups prior to gut casein infusion (Supplementary Table 3). Furthermore, there was no significant difference in plasma insulin or glucagon levels between saline- and casein-infused rats during the IVGTT (Fig. 1e and Supplementary Fig. 1b), suggesting that changes in the levels of these glucoregulatory hormones does not underlie the improvement in glucose tolerance following small intestinal casein infusion.

Previous studies have shown that increased levels of circulating amino acids can both positively and negatively influence glucose homeostasis^37,38^. To confirm that the glucoregulatory role of casein infusion was restricted to the gut, we measured free amino acid levels in circulation. Consistent with previous findings that 20% intralipid administered into the upper small intestine at the same rate/duration does not increase free fatty acid levels^39^, systemic and portal free amino acid levels were comparable between animals that received upper small intestinal infusion of saline or casein (Fig. 1f). To further confirm that the glucoregulatory effect of intestinal casein infusion was preabsorptive, we co-infused the topical anesthetic tetracaine at a dose that has previously been shown to inhibit the ability of preabsorptive intestinal lipid-sensing mechanisms to regulate glucose homeostasis by blocking the neurotransmission of local gut vagal afferent fibers^39^. Infusion of tetracaine alone had no effect on glucose tolerance during the IVGTT (Fig. 1g). However, co-infusion of tetracaine with casein reversed the ability of casein to improve glucose tolerance as observed by the similar elevation in percentage of change in glucose (Fig. 1g) and representative AUC (Fig. 1h) during the IVGTT compared to saline control. Considered altogether, this indicates that preabsorptive upper small intestinal protein-sensing mechanisms regulate whole-body glucose homeostasis in healthy rodents.

### PepT1- and GLP-1-mediated mechanisms lower GP

**Fig. 2:**
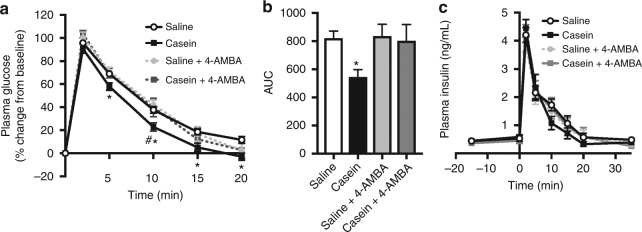
Inhibition of peptide transporter 1 (PepT1) reverses the ability of casein infusion to increase glucose tolerance in healthy rodents. Percentage of change in plasma glucose levels (**a**), integrated area under the curve (AUC, **b**), and plasma insulin levels (**c**) over time during the IVGTT for healthy rats that received an upper small intestinal infusion of saline (*n* = 18), casein (*n* = 24), saline+the competitive PepT1 antagonist 4-aminomethylbenzoic acid (4-AMBA, *n* = 7), or casein+4-AMBA (*n* = 6). Values are presented as mean ± s.e.m., where asterisk (*) represents *p* < 0.05 compared to saline control and hash (^#^) represents *p* < 0.05 compared to casein+4-AMBA (assessed using ANOVA with Tukey post-hoc test)

We next sought to determine the mechanisms through which upper small intestinal casein infusion improves glucose tolerance. Based on the available evidence linking PepT1 activation to gut peptide release in vitro^25,34,35^ and the activation of vagal afferent fibers following in vivo duodenal peptone infusion^30^, we investigated whether PepT1 mediates the glucoregulatory effect of intestinal protein sensing. To do this, we blocked intestinal protein uptake by PepT1 using the non-translocated competitive inhibitor 4-aminomethylbenzoic acid (4-AMBA)^40^. PepT1 inhibition alone had no effect on glucose tolerance as assessed via IVGTT; however, co-infusion of casein and 4-AMBA reversed the ability of casein to improve glucose tolerance 10 min following injection of the glucose bolus (Fig. 2a). Consistent with this, administration of 4-AMBA increased the AUC of casein-infused rats to levels comparable to saline-infused animals (Fig. 2b). Importantly, 4-AMBA treatment did not alter insulin levels compared to saline control (Fig. 2c). Collectively, this indicates that administration of upper small intestinal casein improves glucose tolerance via PepT1-mediated preabsorptive signaling mechanisms.

**Fig. 3:**
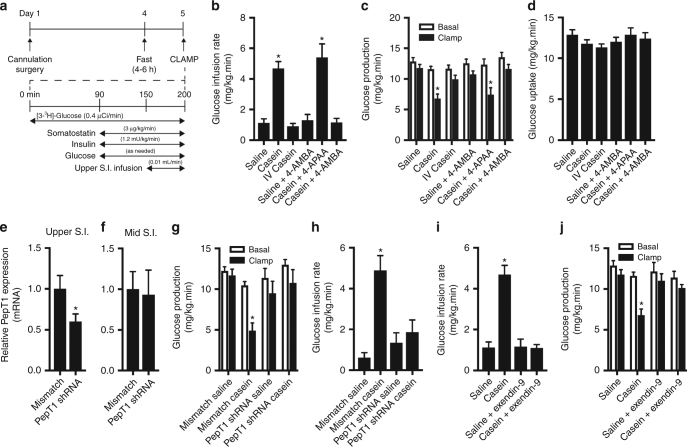
Upper small intestinal casein infusion lowers glucose production through activation of PepT1 in healthy rodents. In vivo glucose kinetics were assessed in conscious, unrestrained healthy rats using the pancreatic (basal insulin) euglycemic clamp as outlined in **a**. Rates of glucose infusion (**b**), glucose production (GP, **c**), and glucose uptake (**d**) in rats that received an upper small intestinal (S.I). infusion of saline (*n* = 10) or casein (*n* = 9), intravenous (IV) infusion of casein (*n* = 5), upper small intestinal infusion of saline+4-AMBA (*n* = 6), casein+4-APAA (*n* = 5), or casein+4-AMBA (*n* = 6). Relative *PepT1* mRNA expression in the mucosal layer isolated from S.I. segments ~6–10 cm (upper S.I., **e**) or ~25–30 cm (mid-S.I., **f**) distal to the pyloric sphincter in rats that received an upper S.I. lentiviral infection with control mismatch (*n* = 6) or *PepT1* shRNA (*n* = 6) particles. Rates of glucose production (**g**) and glucose infusion (**h**) in rats that received mismatch shRNA lentiviral infection+saline infusion (*n* = 6), mismatch+casein infusion (*n* = 6), *PepT1* shRNA lentiviral infection+saline infusion (*n* = 5), or *PepT1* shRNA+casein infusion (*n* = 6). Rates of glucose infusion (**i**) and glucose production (**j**) in rats that received saline+the GLP-1 receptor antagonist exendin-9 (*n* = 3) or casein+exendin-9 (*n* = 6). Values are presented as mean ± s.e.m., where basal represents the average GP of *t* = 60–90, clamp represents the average GP of *t* = 190–200 and asterisk (*) represents *p* < 0.05 compared to saline control. Statistical significance was determined using an unpaired, two-tailed *t*-test (two groups) or ANOVA with Tukey post-hoc test (3+ groups)

The observed improvement in glucose tolerance following intra-upper small intestinal protein infusion in healthy rodents can be accounted for by an increase in glucose uptake or a suppression of GP, although plasma insulin and glucagon levels remain unaltered by casein infusion (Fig. 1e and Supplementary Fig. 1b). To investigate whether intestinal protein administration alters steady-state changes in glucose kinetics through direct effects in the gut, we performed a pancreatic euglycemic clamp and administered upper small intestinal saline or casein at the same dose as in the IVGTT (Fig. 3a), while plasma insulin levels were maintained at basal levels in healthy rats (Supplementary Table 3). Of note, there was no significant impact on in vivo glucose kinetics following intra-upper small intestinal infusion of water compared to saline (Supplementary Fig. 2a–d) and therefore saline was used as a control in all clamp experiments. We found that rats receiving intestinal casein infusion in the clamped setting required a significantly higher (~4.7-fold) rate of exogenous glucose infusion to maintain euglycemia compared to saline control (Fig. 3b). This was associated with decreased levels of GP during the clamp compared to basal (Fig. 3c) or approximately a 42.3 ± 6.3% suppression of GP (versus 8.0 ± 3.6% for saline-infused animals; Supplementary Fig. 2e). Importantly, this was not associated with a change in glucose uptake (Fig. 3d), suggesting that the increase in glucose infusion rate was entirely accounted for by a decrease in GP. Similar to the IVGTT, the influence of casein on GP was independent of any differences in post-surgical body weight or plasma glucose and insulin levels (Supplementary Table 3). Importantly, intravenous administration of casein (at the same dose as administered to the upper small intestine) had no influence on in vivo glucose kinetics during the clamp compared to basal conditions (Fig. 3b–d, Supplementary Fig. 2e). This further confirms that the action of upper small intestinal casein is restricted to the gut. Of note, upper small intestinal casein infusion also increased the glucose infusion rate necessary to maintain euglycemia (~4.4-fold) and decreased the rate of GP under clamped conditions (49.1 ± 7.7% suppression), with no change in glucose uptake, in rats that received an extended period of recovery (Supplementary Fig. 3). Considered altogether, this suggests that upper small intestinal protein sensing directly increases glucose tolerance through a suppression of GP.

To alternatively confirm that PepT1 mediates the improvement in glucose tolerance following upper small intestinal casein infusion (Fig. 2) and that protein-sensing mechanisms decrease GP, we next inhibited PepT1 activity during the clamps. Chemical inhibition of PepT1 following co-infusion of 4-AMBA at the same dose used in the IVGTT had no effect on in vivo glucose kinetics under clamped conditions (Fig. 3b–d and Supplementary Fig. 2e). However, co-infusion of casein and 4-AMBA significantly reduced the glucose infusion rate necessary to maintain euglycemia compared to casein-infused animals (Fig. 3b). This was associated with a restoration of GP comparable to saline- or 4-AMBA-infused rats (Fig. 3c) and no change in glucose uptake (Fig. 3d). Importantly, co-infusion of casein and 4-aminophenylacetic acid (4-APAA), the inactive analog of 4-AMBA, resulted in an increased glucose infusion rate comparable to that of casein alone (5.4-fold, Fig. 3b). This was associated with a reduction in GP (40.3 ± 6.6% suppression) and no change in glucose uptake (Fig. 3c, d, Supplementary Fig. 2e), confirming that the action of 4-AMBA is specific. Collectively, this supports a role for PepT1 activation in the GP-lowering effect of intestinal casein infusion.

To further confirm the glucoregulatory role of upper small intestinal PepT1 activity, we reduced endogenous PepT1 levels using lentiviral-mediated transduction of *PepT1* short hairpin RNA (shRNA). Upper small intestinal *PepT1* knockdown resulted in a 40% reduction in *PepT1* expression compared to mismatch control (Fig. 3e). Importantly, decreased *PepT1* expression was restricted to the upper small intestine (lower duodenum/upper jejunum) as *PepT1* expression was similar between mismatch- and PepT1 shRNA-transduced animals in the mid small intestine (mid-jejunum) (Fig. 3f). Prior to initiation of the gut infusion and the pancreatic clamp, rates of basal GP were similar between mismatch and *PepT1* shRNA-transduced rats (Fig. 3g). However, upper small intestinal casein infusion decreased GP (Fig. 3g and Supplemental Fig. 2f) and consequently increased the glucose infusion rate required to maintain euglycemia (Fig. 3h) in rats that received mismatch control, but not *PepT1* shRNA, compared to saline infusion. Consistent with earlier results, casein infusion had no effect on glucose uptake in rats expressing either mismatch or *PepT1* shRNA in the upper small intestine (Supplementary Fig. 2g). Notably, the finding that PepT1 knockdown reversed the ability of casein to reduce GP highlights that delivery of protein infusate to the upper small intestine was effective, although the physiological relevance of the PepT1 shRNA studies warrants further investigations. Altogether, this strengthens the role of PepT1 in mediating the glucoregulatory effects of protein sensing and indicates that changes in GP are a key mechanism in improving glucose tolerance following upper small intestinal casein infusion.

As previous studies have highlighted the role of PepT1 activation in the stimulation of GLP-1 release^25,35^ and GLP-1 release is associated with beneficial effects on GP and tolerance, we next investigated whether the influence of upper small intestinal casein infusion on GP relies on the action of GLP-1 signaling. Importantly, upper small intestinal infusion of the GLP-1 receptor antagonist exendin-9 alone had no effect on in vivo glucose kinetics (Fig. 3i, j and Supplementary Fig. 2h, i). However, co-administration of casein and exendin-9 restored the rates of glucose infusion and GP comparable to those of rats that received saline control infusion (Fig. 3i, j). This demonstrates that changes in GP mediated via GLP-1 play an essential role in mediating the effect of upper small intestinal casein administration on GP.

### PepT1-mediated mechanisms are physiologically relevant

**Fig. 4:**
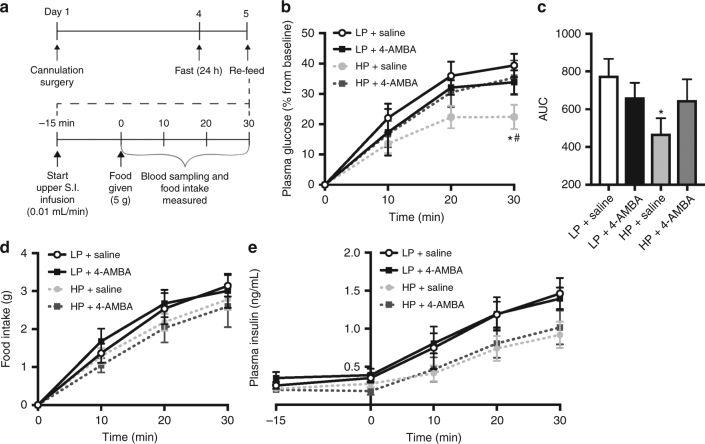
Upper small intestinal PepT1-mediated protein sensing is physiologically relevant during refeeding in healthy rats. Conscious, unrestrained healthy rats underwent a fasting–refeeding protocol in which a low protein (LP) or casein-enriched high protein (HP) diet was offered ad libitum as outlined in **a**. Percentage of change in plasma glucose levels and integrated area under the curve (**b**,** c**), cumulative food intake (**d**), and plasma insulin levels (**e**) were monitored in rats that received LP or HP chow with an upper small intestinal (S.I.) infusion of saline (LP: *n* = 7, HP: *n* = 9) or 4-AMBA (LP: *n* = 6, HP: *n* = 9). Values are presented as mean ± s.e.m., where asterisk (*) represents *p* < 0.05 compared to LP saline and hash (^#^) represents *p* < 0.05 compared to HP+4-AMBA. For the analysis of a single time point, ANOVA with Tukey post-hoc test was used to determine statistical significance

Having confirmed that a PepT1-mediated upper small intestinal protein-sensing mechanism regulates GP and tolerance, we next sought to determine whether this contributes to the physiological regulation of glucose homeostasis following refeeding of a casein-enriched HP diet in healthy rats (Fig. 4a). We first found that rats that received an upper small intestinal saline infusion and were re-fed an isocaloric 60% HP diet following a 24 h fast exhibited lower glucose levels compared to those re-fed a 20% LP diet (Fig. 4b). This was characterized by significantly lower glucose levels 30 min following the initiation of feeding (HP: 22.4 ± 4.0% versus LP: 39.4 ± 3.9% increase from baseline) and was associated with a significant reduction (~60%) in the AUC (Fig. 4c). Importantly, the effect of HP refeeding was independent of changes in food intake (Fig. 4d) but associated with a non-significant trend for decreased plasma insulin levels in rats receiving HP vs. LP (Fig. 4e). To examine whether PepT1-mediated protein-sensing mechanisms contribute to the post-feeding glucose response, we infused 4-AMBA into the upper small intestine for 15 min prior to refeeding and monitored blood glucose levels for 30 min (the equivalent duration of time for which 4-AMBA increased glucose levels following initial nutrient exposure in the IVGTT). While administration of 4-AMBA alone had no influence on the glucose response for LP-fed rats, infusion of 4-AMBA into the upper small intestine of rats re-fed a HP diet resulted in significantly increased plasma glucose levels that were comparable to those of LP-fed rats (Fig. 4b, c). This was independent of changes in cumulative food intake or insulin levels (Fig. 4d, e). Considered altogether, this indicates that upper small intestinal PepT1-mediated protein-sensing mechanisms underlie the acute glucose-lowering effect of HP refeeding and demonstrates the relevance of intestinal protein sensing under healthy physiological conditions.

### Protein-sensing mechanisms remain intact under disease conditions

**Fig. 5:**
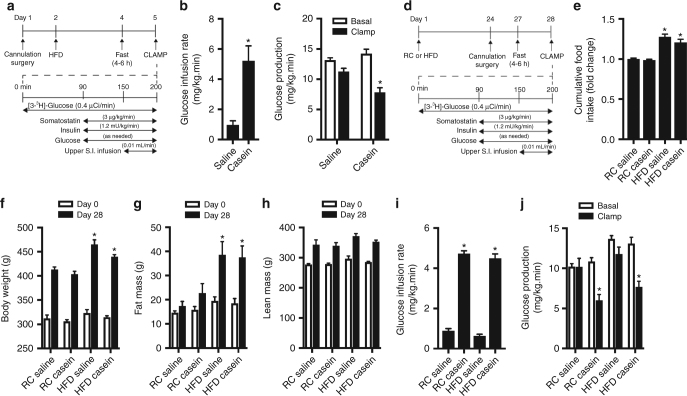
Upper small intestinal infusion of casein lowers glucose production in models of early-onset insulin resistance and obesity. Rats were fed a 3-day high-fat diet (HFD) and a pancreatic (basal insulin) euglycemic clamp was performed in rats as outlined in **a**. Rates of glucose infusion (**b**) and glucose production (GP, **c**) were determined in HFD rats that received an upper small intestinal (S.I.) infusion of saline (*n* = 7) or casein (*n* = 6). Rats were fed a regular chow (RC) or HFD for 28 days and a pancreatic (basal insulin) euglycemic clamp performed as outlined in (**d**). Cumulative food intake was monitored over the 28-day protocol (**e**). Body weight (**f**), fat mass (**g**), and lean mass (**h**) were measured at baseline (day 0) and at the end of the 28-day period. Rates of glucose infusion (**i**) and glucose production (**j**) were assessed following an upper S.I. infusion of saline (*n* = 5–6) or casein (*n* = 5–8). Values are presented as mean ± s.e.m., where basal and clamp represent the average GP of *t* = 60–90 or *t* = 190–200, respectively, and asterisk (*) represents *p* < 0.05 compared to respective saline control (**b**, **c**,** i**, **j**) or regular chow-fed rats (**e**–**g**). Statistical significance was determined using an unpaired, two-tailed *t*-test (two groups) or ANOVA with Tukey post-hoc test (3+ groups)

Given that our data highlight a novel, physiologically relevant protein-sensing pathway that lowers GP and increases glucose tolerance, we ultimately investigated whether activation of upper small intestinal PepT1-mediated protein-sensing mechanisms can reduce blood glucose levels in the context of metabolic disease. We first examined the efficacy of protein-sensing mechanisms in a 3-day high-fat diet (HFD)-fed rodent model of early-onset insulin resistance (Fig. 5a), which has previously been shown to reverse the ability of upper small intestinal lipid-sensing mechanisms to regulate glucose homeostasis^39^. Consistent with previous studies^41^, 3-day HFD-fed rats were hyperphagic (Supplementary Fig. 4a) compared to animals that received regular chow (RC) and exhibited hyperinsulinemia (RC: 1.1 ± 0.4 ng mL^−1^ versus HFD: 1.7 ± 0.6 ng mL^−1^, *p* < 0.05 (two-tailed, unpaired *t*-test)) (i.e., evidence of insulin resistance) despite no difference in post-surgical body weight (Supplementary Fig. 4b). In contrast to intestinal lipid-sensing pathways, we found that upper small intestinal infusion of casein resulted in a requirement for a significantly higher (~5.4-fold) rate of exogenous glucose infusion compared to saline infusion to maintain euglycemia during the pancreatic clamps (Fig. 5b). As observed with healthy rodents, this was associated with a significant decrease in GP, but no change in glucose uptake, compared to saline-infused rats (Fig. 5c and Supplementary Fig. 4c, d). Importantly, this indicates that GP-lowering protein-sensing mechanisms remain intact under conditions of early insulin resistance.

We next examined the ability of upper small intestinal casein infusion to influence in vivo glucose kinetics in a longer experimental protocol whereby rats were fed RC or HFD for 28 days (Fig. 5d). Notably, the 28-day HFD model has previously been shown to induce hepatic and peripheral insulin resistance and obesity^42–44^. Rats fed a HFD were hyperphagic (~30% kcal increase) and exhibited significantly increased body mass compared to rats fed a RC diet over the 28-day period (Fig. 5e, f). This was associated with increased adiposity as demonstrated by significantly higher fat mass but not lean mass as monitored by Echo-magnetic resonance imaging (EchoMRI; Fig. 5g, h). Consistent with the shorter 4-day protocol used in Figs. 3 and 5a–c, rats fed a RC diet for 28 days that received an upper intestinal infusion of casein required a significantly higher glucose infusion rate (~4.7-fold) to maintain euglycemia under the clamped setting compared to rats that received saline (Fig. 5i). This was associated with 43.5 ± 7.9% suppression of GP compared to basal conditions (Fig. 5j and Supplementary Fig. 4e) and no change in glucose uptake (Supplementary Fig. 4f). Importantly, while an upper intestinal infusion of saline did not influence in vivo glucose kinetics in rats fed a 28-day HFD, rats that received an upper small intestinal casein infusion during the clamp required a higher glucose infusion rate (~4.5-fold) to prevent hypoglycemia (Fig. 5i). Similar to healthy and 3-day HFD-fed rats, this chronic obese model was associated with a 39.6 ± 6.6% decrease in GP (Fig. 5j and Supplementary Fig. 4e) and no change in glucose uptake (Supplementary Fig. 4f). Altogether, this demonstrates that upper small intestinal protein-sensing mechanisms are conserved in a model of long-term insulin resistance and obesity.

**Fig. 6:**
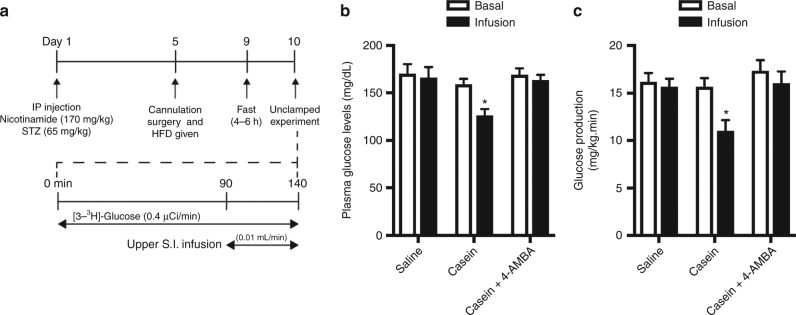
Upper small intestinal infusion of casein reduces plasma glucose levels and is associated with a reduction in glucose production in hyperglycemic rodents. A non-pancreatic clamp experiment was performed in rats subjected to nicotinamide–streptozotocin (STZ)/HFD-induced hyperglycemia as outlined in **a**. Plasma glucose levels (**b**) and GP (**c**) were assessed in hyperglycemic rats receiving an upper S.I. infusion of saline (*n* = 6), casein (*n* = 7), or casein+4-AMBA (*n* = 6). Values are presented as mean ± s.e.m., where basal and infusion represent the average GP of *t* = 60–90 or *t* = 120–140, respectively, and asterisk (*) represents *p* < 0.05 compared to saline control. Statistical significance was determined using ANOVA with Bonferonni post-hoc test

To further investigate the therapeutic relevance of intestinal protein-sensing mechanisms, we examined whether upper small intestinal protein administration influences glucose homeostasis in the nicotinamide–streptozotocin–HFD-induced (NA/STZ-HFD) hyperglycemic rat model (Fig. 6a). Of note, NA/STZ-HFD rats exhibited increased basal GP and plasma glucose levels in the presence of comparable plasma insulin levels to healthy rats (Supplementary Fig. 5a–c) as previously described^42,43,45^. Under non-clamped conditions, direct upper small intestinal infusion of casein in NA/STZ-HFD-induced hyperglycemic rats for the same 50 min interval as during the IVGTT and clamp resulted in a significant decrease in plasma glucose levels (20.8% suppression) compared to saline infusion (2.3% suppression, Fig. 6b). This reduction in plasma glucose levels was associated with a 28.3% decrease in GP (Fig. 6c and Supplementary Fig. 5d), further highlighting that unlike intestinal lipid-sensing mechanisms, protein-sensing pathways able to suppress GP remain functional in the context of metabolic disease. Importantly, co-infusion of 4-AMBA reversed the ability of casein to lower plasma glucose levels and reduce GP in NA/STZ-HFD rats (Fig. 5b, c and Supplementary Fig. 5d). This confirms the glucoregulatory importance of PepT1 activation and demonstrates for the first time that upper small intestinal PepT1 is a novel therapeutic target for lowering blood glucose levels.

## Discussion

In the current study, we demonstrate for the first time that protein sensing in the upper small intestine improves glucose homeostasis in healthy, obese, and hyperglycemic rodents. Previous studies have postulated several mechanisms whereby HP intake can improve glucose tolerance that include increased insulin secretion, an exchange of carbohydrate for protein in the diet, and a lower gastric-emptying rate that would slow the appearance of glucose into circulation^46^. Additionally, increased protein intake has been shown to initiate a portal gut–brain axis mediated via the activation of μ-opiod receptors to increase intestinal gluconeogenesis and influence metabolic parameters, such as food intake^47^. While our findings cannot exclude these possibilities, our observations that inhibition of PepT1 influenced post-prandial glucose levels when rats consumed the same amount of HP as PepT1-intact rats (Fig. 4) and that the glucose-lowering effect of upper small intestinal protein administration under physiological conditions was not secondary to increases in circulating insulin or glucagon levels (Figs. 1 and 4) suggests that protein action in the gut is not secondary to diet content nor changes in glucoregulatory hormone levels. Furthermore, upper small intestinal protein administration improved glucose tolerance following an intravenous injection of glucose, thereby removing the variable of gastric emptying. As both systemic and portal amino acid levels were comparable between saline- and casein-infused rats (Fig. 1f) and intravenous administration of casein given at an identical equimolar dose as upper small intestinal infusion had no influence on in vivo glucose kinetics (Fig. 3b–d), the available evidence supports the notion that preabsorptive protein action in the upper small intestine directly stimulates pathways that regulate whole-body glucose homeostasis.

Our finding that intestinal protein-sensing mechanisms lower blood glucose levels through a suppression of GP are similar to those previously described for upper small intestinal lipid sensing^39^. The observation that both protein and lipid can regulate glucose homeostasis through preabsorptive signaling pathways highlights the glucoregulatory importance of rapid and potent negative feedback initiated at the level of the gut. Mechanistically, lipid-sensing pathways trigger intracellular signaling cascades that stimulate the exocytosis of gut peptides such as CCK, and the subsequent gut peptide-mediated activation of local vagal afferent neurons initiates a gut–brain axis to regulate glycemia (for a review, see ref. ^48^). Given that both protein administration and PepT1 activation stimulate gut peptide release and our observation that co-infusion of the anesthetic tetracaine reverses the ability of intestinal casein to improve glucose tolerance, we hypothesized that upper small intestinal protein sensing also improves glucose tolerance by a gut-peptide-mediated neuronal network. As high-fat feeding rapidly impairs the ability of intestinal lipid sensing to suppress GP via resistance acquired at the level of CCK signaling at the CCK1 receptor^39,49^ and protein-sensing mechanisms remained intact under disease conditions (Figs. 5 and 6), we investigated the possibility that CCK1 receptor-independent pathways mediate the glucoregulatory effect of intestinal protein sensing. Indeed, we demonstrated that administration of the GLP-1R antagonist, exendin-9, reversed the ability of casein to decrease GP, thus highlighting the contribution of gut GLP-1 signaling to the glucoregulatory role of intestinal casein infusion. Our data are consistent with previous reports which demonstrate that PepT1 activation stimulates GLP-1 release^25,35^. However, it is also possible that other gut peptides contribute to the glucose-lowering effect of upper small intestinal protein administration. These could include CCK, which was not addressed in the current study, or other gut peptides. For instance, PYY has been shown to mediate the effect of dietary protein intake on metabolic parameters, including satiety and adiposity^50^, and future studies that investigate the contribution of PYY and other gut peptides in relation to the glucoregulatory role of intestinal protein-sensing mechanisms are warranted.

 Of note, a previous report suggests that intestinal protein is more potent than equicaloric amounts of lipid or carbohydrate to stimulate gut peptide GLP-1 and PYY release^29^. Consistent with this, our findings revealed that a lower dose of casein hydrolysate (0.32 kcal mL^−1^) was required to reduce GP to the same extent as lipid infusion (2.0 kcal mL^−1^)^39^. In addition, in the current study we administered casein hydrolysate, which is a soluble source of polypeptide, peptides and free amino acids. Given that PepT1 is an exclusive di- and tri-peptide transporter^51^, this suggests that di- or tri-peptides, versus free amino acids, are responsible for the observed glucoregulatory effects. Therefore, it remains to be explored whether, similar to the requirement for the uptake and metabolism of intestinal lipids to fatty acyl-CoA^39^, intracellular metabolism of peptides is necessary to activate gut-mediated signaling pathways to lower GP. Alternatively, evidence suggests that activation of PepT1 by a non-metabolizable substrate is sufficient to trigger membrane depolarization and stimulate GLP-1 release^25^. To elucidate key differences and similarities between lipid and protein-sensing mechanisms, future studies that investigate the downstream mechanisms of PepT1-activated signaling are essential.

Using both molecular and chemical approaches, this work highlights a novel metabolic role of PepT1 in the upper small intestine. PepT1 knockout mice have reduced intestinal uptake of peptide but are otherwise viable, fertile and exhibit normal body weight on a RC diet. Interestingly, both increased dietary protein^52^ and short-term fasting enhance PepT1 expression^53,54^. It has been proposed that PepT1 upregulation occurs in preparation to efficiently transport peptides during refeeding but evidence also suggests that Pept1 is only required for amino acid absorption during HP intake^55^. In support of this, our finding that inhibition of PepT1 influenced the post-prandial glucose response following HP but not LP refeeding suggests that the contribution of PepT1 action to glucose homeostasis is negligible under basal conditions but plays an important role in a post-prandial setting following HP intake. Notably, starvation induces Pept1 expression and therefore subsequent absorption capacity to the greatest extent in the upper small intestine^56,57^, highlighting the biological relevance of upper small intestinal protein-sensing mechanisms under physiological refeeding conditions. While we cannot exclude that HP refeeding reduced blood glucose levels due to actions on peripheral tissues such as the kidney or a difference in carbohydrate intake, the observation that local intestinal 4-AMBA administration inhibited the reduction in blood glucose levels compared to rats that received saline (where the effect on HP on peripheral tissues would be comparable) indicates that local upper small intestinal PepT1 action contributes to the glucose-lowering effect of increased protein dietary content. Of note, a 40% reduction in *PepT1* mRNA was sufficient to completely abolish the ability of casein infusion to decrease GP. PepT1 activation has been demonstrated to trigger membrane depolarization, opening of voltage-gated Ca^2+^ currents and subsequent GLP-1 release^25^. As we demonstrated that GLP-1 signaling contributed to the glucoregulatory role of intestinal casein infusion, we hypothesize that such a reduction in PepT1 expression does not allow for the threshold of Ca^2+^ channel opening necessary for depolarization, and subsequent GLP-1 release, to be reached. This is consistent with a previous study that demonstrated acute (41%) knockdown of protein kinase Cζ in the rat ileum inhibited oleic acid-induced GLP-1 release^58^. However, future studies that investigate the mechanism underlying GLP-1 release and signaling in response to PepT1 activation are warranted. Notably, the use of genetically modified rodents, such as the Pept1 knockout mouse or generation of inducible and/or upper small intestinal-specific PepT1 knockout models, will be beneficial to further characterize the identified PepT1–GLP-1-dependent protein-sensing pathway. Finally, it is important to note that other amino acid- or peptide-sensing receptors, such as the calcium-sensing receptor, the taste type 1/3 receptor dimer and GPRC6A, or transporters, such as GPR93, μ-opiod receptors or transporters, may also contribute to the glucose-lowering effect of intestinal casein administration, and studies that further investigate the downstream mechanisms will be valuable.

A key finding of this study is that the identified PepT1-dependent pathway is functional in insulin-resistant, obese, and hyperglycemic rodents, highlighting the unique therapeutic potential and translational relevance of intestinal protein-sensing mechanisms. PepT1 membrane expression and function is regulated by various parameters, including development^59^, diurnal rhythm/feeding cycles^60^, hormones such as insulin^61^ and leptin^62^, and inflammatory intestinal disease^63^. While previous reports demonstrate that PepT1 expression is decreased in enteroendocrine cells isolated from mice fed a HFD and that small intestinal PepT1 expression and function is decreased in rodent models of obesity and type 1 diabetes^64–66^, other studies have shown that PepT1 localization and activity at the brush-border membrane increases in rodent models of type 2 diabetes with hyperinsulinemia^66,67^. Importantly, our findings suggest that, despite reported changes in expression and activity, PepT1 is a viable target under conditions of metabolic disease. This suggests that exploiting the glucose-lowering ability of intestinal protein sensing is therapeutically valuable compared to lipid mechanisms, where it is necessary to bypass blocks in the glucose-lowering pathways as resistance is acquired. Additionally, previous studies have shown negative consequences of increased circulating amino acid levels on insulin resistance in peripheral tissues^37,38^, altogether indicating that directly targeting upper small intestinal preabsorptive protein-sensing mechanisms will allow for the exploitation of the beneficial effects on glucose homeostasis. Given that pharmacological targeting of gut-localized signaling pathways via metformin has relevance in the treatment of metabolic disease^42,43,68^, this further supports the development of therapeutics that target intestinal protein-sensing pathways to improve glucose homeostasis.

Considered altogether, our current finding that upper small intestinal protein sensing regulates glucose tolerance in healthy and obese/diabetic rodents not only improves our understanding of how HP intake improves glucose homeostasis but also furthers our understanding of the physiological relevance of intestinal nutrient-sensing pathways. Importantly, this work provides the foundation for the development of novel therapeutic strategies targeting PepT1-mediated intestinal protein-sensing mechanisms to reduce blood glucose levels in metabolic disease.

## Methods

### Animals

All animal protocols were reviewed and approved by the Institutional Animal Care and Use Committee at the University Health Network in accordance with the Canadian Council on Animal Care guidelines. Male Sprague-Dawley rats (250–270 g upon arrival, approximately 8 weeks of age) were obtained from Charles River Laboratories (Montreal, QC, Canada) and were allowed to acclimatize for 5–6 days before manipulation. Rats were housed in a 12-h dark:light cycle with free access to regular rat chow (Teklad 7002, Harlan Laboratories, Madison, WI) and drinking water. Rats were randomly assigned into various diet and treatment groups as described below and no rats were excluded unless otherwise indicated. Sample size was chosen based on previously published experiments performed under similar conditions. The experimenter was not blinded to the experimental conditions. The nutritional composition of various diets used in the experiments described below are displayed in Supplementary Table 1.

### Surgical procedure and recovery

Rats were anesthetized with an intraperitoneal injection of ketamine (Vetalar, Bioniche, Belleville, ON) and xylazine (Rompun, Bayer, Toronto, ON). For insertion of the gut catheter, a 4-cm incision was made along the ventral midline, the duodenum isolated, and a small hole made in the intestinal wall approximately 6 cm distal from the pyloric sphincter. An intestinal cannula was inserted to target the lower duodenum and upper jejunum and secured to the outer surface of the intestine using tissue adhesive (3M Vetbond, London, ON). The cannula was tunneled subcutaneously from the abdomen, exiting through an incision in the back of the neck rostral to the interscapular area, and the abdominal wall closed. The gut line was flushed each day with 0.1 mL of saline and sealed with a metal pin to ensure patency. A subset of rats received upper small intestinal injections of purified lentiviral particles expressing mismatch or rat PepT1 shRNA (1 × 10^6^ infection units, Santa Cruz, Dallas, TX, USA), which has previously been optimized to specifically infect the upper and not the lower small intestine^42^. The upper small intestine was tied and closed with 4–0 sutures approximately 6-12 cm distal to the pyloric sphincter (to target the same region as the infusion protocol), and the contents were washed out with saline. Subsequently, a 1:10 dilution of lentiviral particles in saline was injected (200 μL total) and incubated for 20 min, following which the sutures were removed and the gut line was placed as described above.

After insertion of the intestinal cannula, catheters were inserted into the carotid artery and jugular vein for blood sampling and intravenous infusion purposes, respectively. Vascular lines were tunneled subcutaneously to the back of neck, filled with 10% heparin, and secured with a metal pin until the time of experiment. Rats were individually housed and received regular chow diet during the recovery period unless indicated otherwise. Body weight and food intake were monitored daily and rats that did not recover were excluded from the study.

### 3-day HFD-induced insulin-resistant model

The 3-day HFD model has previously been shown to induce hepatic and hypothalamic insulin resistance and upper small intestinal lipid-sensing defects^39,42–44,49^. The day following surgery, rats were given free access to a 10% lard-enriched HFD (Test Diet, St. Louis, MO, USA). Food intake was monitored daily and rats that were hyperphagic and consumed more calories than rats receiving regular chow were included in the study.

### 28-day HFD-induced obese model

The 28-day HFD model has previously been shown to induce hepatic and peripheral insulin resistance and obesity^42–44^. Rats were given free access to a 10% lard-enriched HFD for 24 days prior to undergoing vascular and gut cannulation surgery. Following surgery, rats were maintained on HFD until clamp experiments. Food intake, body weight, and body fat mass was monitored weekly. Analysis of body mass composition in conscious rats was obtained using the EchoMRI body composition analyzer (EchoMRI, Houston, TX) as recommended by the manufacturer. As a control, a separate group of rats were fed RC diet for 28 days.

### STZ/NA-HFD-induced model of hyperglycemia

Induction of a diabetic rat model with moderate and stable hyperglycemia but no compensatory increase in plasma insulin levels^42,43,45^ was performed. Rats were injected with NA (170 mg kg^−1^, Sigma Aldrich, Oakville, ON) and STZ (65 mg kg^−1^, Sigma Aldrich) intraperitoneally 15 min apart. Four days following injection, rats underwent gut and vascular cannulation surgery and were given HFD until the study was performed 5–6 days later. Rats with plasma glucose levels >140 mg dL^−1^ on the day of experimentation were considered hyperglycemic and included in the study. Rats with plasma glucose levels >300 mg dL^−1^ were excluded from the study.

### Intestinal infusions and treatments

Intestinal treatments were continuously infused into the upper small intestine through the gut cannula for 1 min at 0.12 mL min^−1^ (to fill the dead space of the cannula) followed by 0.01 mL min^−1^ for a total of 50 min. The timing of this protocol was based on previous studies with upper small intestinal fatty acid infusion whereby the effect was preabsorptive with no leak into portal or systemic circulation^39^. Gut treatments were administered using a PHD 2000 infusion pump (Harvard Apparatus, Holliston, MA). In all experiments, 8% (w/v) casein solution (Sigma Aldrich, Oakville, ON) was prepared by solubilizing casein in distilled water by titrating to a pH of 1 with 10 N HCl followed by the addition of 10 N NaOH to a final pH of 5.0 (the native pH of the rat duodenum under fed conditions^36^). The casein solution was made fresh 5–10 min before each experiment and therefore the pH was measured prior to every infusion. The typical amino acid content of casein is shown in Supplementary Table 2. The casein dose was optimized using a dose–response curve and is similar to studies that show stimulation of activation of vagal afferent firing by 8% peptone^30^. Saline (0.9%, Baxter, Mississauga, ON) or water (titrated similarly to the casein solution, pH 5.0) were infused as control treatments. Tetracaine (5.67 mM, Sigma Aldrich), the competitive PepT1 inhibitor 4-AMBA (20 mM, Sigma Aldrich), the inactive analog 4-APAA (20 mM, Sigma Aldrich) and exendin-9 (15 μg mL^−1^, Tocris Bioscience, Ellisville, MO, USA) were co-infused with saline or casein solutions as indicated. Tetracaine was infused at a dose previously established to block preabsorptive intestinal lipid sensing^39^, and the concentration of 4-AMBA was initially based on a dose previously shown to decrease activation of duodenal vagal afferent fiber activation^30^ and then optimized using a dose–response experiment. The dose of exendin-9 used was previously shown to inhibit the effects of ileal fatty acid sensing^69^.

### Intravenous glucose tolerance test

Rats were fasted 16 h prior to performing the IVGTT (fast initiated at 5:00 P.M.). IVGTT experiments were performed in conscious, unrestrained rats 4 days post-surgery. The intestinal infusion was initiated at *t* = −15 min and rats received an intravenous bolus of glucose (0.25 g kg^−1^, 20% glucose stock solution, Sigma Aldrich, Oakville, ON) over 10 s via the jugular vein cannula at *t* = 0. Blood samples were collected at *t* = −15 min (initiation of gut infusion), 0 (prior to glucose injection), 2, 5, 10, 15, 20, 25, 30, and 35 min for plasma glucose and amino acid analyses. At *t* = 35 min, rats were anesthetized with intravenous ketamine and a portal blood sample was collected.

### Pancreatic (basal insulin) euglycemic clamp

Basal insulin euglycemic pancreatic clamps were performed in conscious, unrestrained rats 4 days or 7–8 days (Supplementary Fig. 3) following surgery. Rats were fasted for 4–6 h prior to the experiment to ensure equivalent post-prandial absorptive nutritional status. On the day of the experiment (initiated at 9:00 A.M.), rats received a continuous intravenous infusion of [3-^3^H]-glucose tracer (40 μCi bolus, 0.4 μCi min^−1^, Perkin Elmer, Woodbridge, ON) for the duration of the experiment (*t* = 0–200 min) to assess glucose kinetics based on the tracer-dilution methodology. Basal glucose kinetics were assessed once the [3-^3^H]-glucose reached steady state by collecting plasma samples every 10 min from *t* = 60–90 min. At *t* = 90 min, the clamp was initiated through continuous intravenous infusion of somatostatin (3 μg kg^−1^ min^−1^; Bachem, Torrance, CA, USA) and insulin was replaced to basal levels (1.2 mU kg^−1^ min^−1^, porcine insulin, Sigma Aldrich, Oakville, ON). An exogenous solution of 25% glucose solution (Sigma Aldrich) was infused at a variable rate as required to maintain euglycemia consistent with basal glucose levels. Infusion of upper intestinal treatments was performed for the final 50 min of the clamp (*t* = 150–200 min) as described above. All infusions were administered using PHD 2000 infusion pumps (Harvard Apparatus, Holliston, MA). Blood samples were collected every 10 min throughout the clamp to determine the specific activity of [3-^3^H]-glucose and measure plasma insulin levels. For the pancreatic clamp/tracer-dilution data analysis, the “basal” GP represents the average from *t* = 60–90 min and “clamp” represents the average GP from *t* = 180–200 min. At *t* = 200, rats were anesthetised with an intravenous administration of ketamine and a portal blood sample was collected. Tissue samples were collected in phosphate-buffered saline containing complete EDTA-free protease inhibitor cocktail (Roche, Mannheim, Germany), snap-frozen in liquid nitrogen, and stored at −80 °C until further analysis.

### Fasting–refeeding experiments

Fasting–refeeding experiments were performed in conscious, unrestrained healthy rats using a protocol previously established by LaPierre et al.^13^. Five days following surgery, rats were subjected to a 24-h fast (initiated at 9:00 A.M.). The following day, basal glucose levels were determined and rats received a 15-min preinfusion of upper small intestinal treatment as described above that continued for the duration of the experiment. At *t* = 0, rats were presented with ~5 g of isocaloric low (20%) or high (60%) protein chow diet with casein as the protein source (TD.91352 and TD.06220, respectively, Harlan Laboratories, Indianapolis, IN, USA) and free access to drinking water. Blood samples were collected at 10-min intervals for analysis of plasma glucose levels and hormone levels. Remaining food was measured every 10 min to calculate cumulative food intake.

### [3-^3^H]-glucose infusion protocol (non-clamped conditions)

Unclamped experiments were performed 4–5 days following surgery in conscious, unrestrained rats based on a protocol previously established^42,43^. Rats were fasted 4–6 h prior to initiation of experiment. On the day of the experiment (initiated at 9:00 A.M.), rats received a continuous infusion of [3-^3^H]-glucose tracer (40 μCi bolus, 0.4 μCi min^−1^, Perkin Elmer, Woodbridge, ON) starting at *t* = 0 min and maintained until the end of the experiment (*t* = 140 min) to assess glucose kinetics under steady-state conditions using the tracer-dilution methodology. Following determination of basal glucose kinetics from *t* = 60–90 min, the intestinal infusion was administered for 50 min as described above (*t* = 90–140 min). Blood samples were collected every 10 min to measure plasma glucose levels and determine the specific activity of [3-^3^H]-glucose. For the analysis of glucose kinetics, the “basal” GP represents the average from *t* = 60–90 min and “infusion” represents the average GP from *t* = 120–140 min.

### Biochemical analysis

Blood samples were collected at the indicated time points in heparinized tubes and centrifuged at 2000 × *g* for 1 min. Plasma glucose levels were determined immediately using the glucose oxidase method using a GM9 glucose analyzer (Analox Instruments, Stourbridge, UK). Remaining plasma was stored at −20 °C until further analysis. For analysis of plasma hormone levels, plasma was stored in tubes containing SigmaFast protease inhibitor cocktail (Sigma Aldrich, Oakville, ON).

Plasma hormone levels were assessed using a rat insulin or glucagon radioimmunoassay kit (EMD Millipore, Billerica, MA, USA) as per the manufacturer’s instructions.

Plasma amino acid levels were assessed using a colorimetric ninhydrin reaction adapted from Matthews et al.^70^. Fifty μL of plasma was diluted 1:4 in distilled water and deproteinized via the addition of an equal volume of 10% (w/v) sodium tungstate (Sigma-Aldrich) and 2 N HCl with successive mixing. After 5 min, samples were centrifuged at 13,000 rpm for 5 min at 4 °C. Two hundred and fifty μL of supernatant was then combined with 125 μL of cyanide-acetate buffer (2.8 mM sodium acetate, 7.2% glacial acetic acid, 26.8 μM disodium EDTA, and 4% cyanide solution, pH 5.2). One hundred and twenty-five μL of ninhydrin reagent (3% (w/v) ninhydrin in 2-methoxyethanol) was then added, and tubes were mixed and boiled. After 15 min, 3.75 mL cold diluent (50% isopropanol in water) was added, and samples were mixed and transferred to 1 cm cuvettes. Absorbance was read using a Genesys 10S VIA spectrophotometer (ThermoScientific, Toronto, ON) at 570 nm. Plasma α-N was calculated by comparing the absorbance of the unknown plasma sample to a known concentration of α-nitrogen.

### Quantitative PCR (qPCR) analysis

Approximately 75 mg of the mucosal layer from upper (~6–10 cm distal from the pyloric sphincter, to contain both lower duodenum and upper jejunum) or lower (~25–30 cm distal from pyloric sphincter, mid-jejunum) small intestinal samples was separated from the smooth muscle layer immediately following dissection. Mucosal scrapings were homogenized in lysis buffer (Ambion) using a PowerGen-125 homogenizer (Thermo Fisher Scientific, Toronto, ON) and centrifuged at 12,500 × *g* for 5 min, and RNA was isolated using the Ambion PureLink RNA Mini Kit per kit guidelines (Thermo Fisher Scientific). RNA was quantified by measuring the absorbance at 260 and 280 nm using Cytation 5 imaging reader (BioTek, Winooski, VT). Four μg of RNA was subjected to DNase digestion (Roche, Mannheim, Germany) at room temperature for 10 min and terminated by the addition of 2.3 mM EDTA and incubating at 70 °C for 15 min. cDNA was generated using the SuperScript Vilo cDNA Synthesis Kit as per the manufacturer’s instructions (Invitrogen, Carlsbad, CA, USA). qPCR was performed using 100 ng of cDNA, TaqMan Gene Expression master mix, and TaqMan primers for rat 18s or PepT1 (Thermo Fisher Scientific) using a QuantStudio 7 Flex qPCR machine (Applied Biosystems). Relative gene expression was calculated using the ΔΔCt method where each sample was normalized to 18s as the reference gene.

### Statistical analysis

All statistical analysis was performed using GraphPad Prism (version 7.0b, GraphPad, La Jolla, CA, USA). All parametric data are shown as mean ± s.e.m. For comparisons at a single timepoint, a two-tailed unpaired Student’s *t*-test (two groups) or one-way analysis of variance (ANOVA) with Tukey post-hoc test (3+ groups) was performed. Measurements performed over time were analyzed with a two-way ANOVA with repeated measures and groups were compared using Bonferonni post-hoc test. For all statistical tests, a significant difference was considered at *p* < 0.05.

### Data availability

All the relevant data are available from the authors on request and/or are included within the manuscript and the Supplementary Information files.

## Electronic supplementary material

Supplementary Information(PDF 493 kb)

## References

[1] World Health Organization. *Global Report on Diabetes*. Available at http://apps.who.int/iris/bitstream/10665/204871/1/9789241565257_eng.pdf (World Health Organization, Geneva, 2016).

[2] Arciero PJ (2008). Moderate protein intake improves total and regional body composition and insulin sensitivity in overweight adults. Metabolism.

[3] Boden G, Sargrad K, Homko C, Mozzoli M, Stein TP (2005). Effect of a low-carbohydrate diet on appetite, blood glucose levels, and insulin resistance in obese patients with type 2 diabetes. Ann. Intern. Med..

[4] Lacroix M (2004). A long-term high-protein diet markedly reduces adipose tissue without major side effects in Wistar male rats. Am. J. Physiol. Regul. Integr. Comp. Physiol..

[5] Pichon L, Huneau JF, Fromentin G, Tome D (2006). A high-protein, high-fat, carbohydrate-free diet reduces energy intake, hepatic lipogenesis, and adiposity in rats. J. Nutr..

[6] Skov AR, Toubro S, Ronn B, Holm L, Astrup A (1999). Randomized trial on protein vs carbohydrate in ad libitum fat reduced diet for the treatment of obesity. Int J. Obes. Relat. Metab. Disord..

[7] Gannon MC, Nuttall FQ, Saeed A, Jordan K, Hoover H (2003). An increase in dietary protein improves the blood glucose response in persons with type 2 diabetes. Am. J. Clin. Nutr..

[8] Blouet C (2006). The reduced energy intake of rats fed a high-protein low-carbohydrate diet explains the lower fat deposition, but macronutrient substitution accounts for the improved glycemic control. J. Nutr..

[9] Calbet JA, MacLean DA (2002). Plasma glucagon and insulin responses depend on the rate of appearance of amino acids after ingestion of different protein solutions in humans. J. Nutr..

[10] Claessens M, Calame W, Siemensma AD, van Baak MA, Saris WH (2009). The effect of different protein hydrolysate/carbohydrate mixtures on postprandial glucagon and insulin responses in healthy subjects. Eur. J. Clin. Nutr..

[11] Day JL (1978). Factors governing insulin and glucagon responses during normal meals. Clin. Endocrinol. (Oxf.).

[12] Gannon MC, Nuttall FQ (1987). Acute effects of ingestion of carbohydrate, protein, or fat on cardiac glycogen metabolism in rats. Metabolism.

[13] LaPierre MP, Abraham MA, Yue JT, Filippi BM, Lam TK (2015). Glucagon signalling in the dorsal vagal complex is sufficient and necessary for high-protein feeding to regulate glucose homeostasis in vivo. EMBO Rep..

[14] Pillot B, Soty M, Gautier-Stein A, Zitoun C, Mithieux G (2009). Protein feeding promotes redistribution of endogenous glucose production to the kidney and potentiates its suppression by insulin. Endocrinology.

[15] Gannon MC, Nuttall FQ (2004). Effect of a high-protein, low-carbohydrate diet on blood glucose control in people with type 2 diabetes. Diabetes.

[16] Gannon MC, Nuttall FQ, Neil BJ, Westphal SA (1988). The insulin and glucose responses to meals of glucose plus various proteins in type II diabetic subjects. Metabolism.

[17] Manders RJ (2005). Co-ingestion of a protein hydrolysate and amino acid mixture with carbohydrate improves plasma glucose disposal in patients with type 2 diabetes. Am. J. Clin. Nutr..

[18] Nuttall FQ, Mooradian AD, Gannon MC, Billington C, Krezowski P (1984). Effect of protein ingestion on the glucose and insulin response to a standardized oral glucose load. Diabetes Care.

[19] Akhavan T, Luhovyy BL, Brown PH, Cho CE, Anderson GH (2010). Effect of premeal consumption of whey protein and its hydrolysate on food intake and postmeal glycemia and insulin responses in young adults. Am. J. Clin. Nutr..

[20] Gunnerud UJ, Heinzle C, Holst JJ, Ostman EM, Bjorck IM (2012). Effects of pre-meal drinks with protein and amino acids on glycemic and metabolic responses at a subsequent composite meal. PLoS ONE.

[21] Ryan AT (2013). Effects of intraduodenal lipid and protein on gut motility and hormone release, glycemia, appetite, and energy intake in lean men. Am. J. Clin. Nutr..

[22] Steinert RE (2014). Effects of intraduodenal infusion of L-tryptophan on ad libitum eating, antropyloroduodenal motility, glycemia, insulinemia, and gut peptide secretion in healthy men. J. Clin. Endocrinol. Metab..

[23] Cordier-Bussat M (1997). Peptones stimulate cholecystokinin secretion and gene transcription in the intestinal cell line STC-1. Endocrinology.

[24] Cordier-Bussat M (1998). Peptones stimulate both the secretion of the incretin hormone glucagon-like peptide 1 and the transcription of the proglucagon gene. Diabetes.

[25] Diakogiannaki E (2013). Oligopeptides stimulate glucagon-like peptide-1 secretion in mice through proton-coupled uptake and the calcium-sensing receptor. Diabetologia.

[26] Pais R, Gribble FM, Reimann F (2016). Signalling pathways involved in the detection of peptones by murine small intestinal enteroendocrine L-cells. Peptides.

[27] Bowen J, Noakes M, Clifton PM (2006). Appetite regulatory hormone responses to various dietary proteins differ by body mass index status despite similar reductions in ad libitum energy intake. J. Clin. Endocrinol. Metab..

[28] Lejeune MP, Westerterp KR, Adam TC, Luscombe-Marsh ND, Westerterp-Plantenga MS (2006). Ghrelin and glucagon-like peptide 1 concentrations, 24-h satiety, and energy and substrate metabolism during a high-protein diet and measured in a respiration chamber. Am. J. Clin. Nutr..

[29] van der Klaauw AA (2013). High protein intake stimulates postprandial GLP1 and PYY release. Obes. (Silver Spring).

[30] Darcel NP, Liou AP, Tome D, Raybould HE (2005). Activation of vagal afferents in the rat duodenum by protein digests requires PepT1. J. Nutr..

[31] Eastwood C, Maubach K, Kirkup AJ, Grundy D (1998). The role of endogenous cholecystokinin in the sensory transduction of luminal nutrient signals in the rat jejunum. Neurosci. Lett..

[32] Faipoux R, Tome D, Gougis S, Darcel N, Fromentin G (2008). Proteins activate satiety-related neuronal pathways in the brainstem and hypothalamus of rats. J. Nutr..

[33] Groneberg DA, Doring F, Eynott PR, Fischer A, Daniel H (2001). Intestinal peptide transport: ex vivo uptake studies and localization of peptide carrier PEPT1. Am. J. Physiol. Gastrointest. Liver Physiol..

[34] Matsumura K, Miki T, Jhomori T, Gonoi T, Seino S (2005). Possible role of PEPT1 in gastrointestinal hormone secretion. Biochem. Biophys. Res. Commun..

[35] Zietek T, Rath E, Haller D, Daniel H (2015). Intestinal organoids for assessing nutrient transport, sensing and incretin secretion. Sci. Rep..

[36] McConnell EL, Basit AW, Murdan S (2008). Measurements of rat and mouse gastrointestinal pH, fluid and lymphoid tissue, and implications for in-vivo experiments. J. Pharm. Pharmacol..

[37] Tremblay F (2005). Overactivation of S6 kinase 1 as a cause of human insulin resistance during increased amino acid availability. Diabetes.

[38] Tremblay F, Marette A (2001). Amino acid and insulin signaling via the mTOR/p70 S6 kinase pathway. A negative feedback mechanism leading to insulin resistance in skeletal muscle cells. J. Biol. Chem..

[39] Wang PY (2008). Upper intestinal lipids trigger a gut-brain-liver axis to regulate glucose production. Nature.

[40] Meredith D (1998). 4-Aminomethylbenzoic acid is a non-translocated competitive inhibitor of the epithelial peptide transporter PepT1. J. Physiol..

[41] Yue JT (2016). Inhibition of glycine transporter-1 in the dorsal vagal complex improves metabolic homeostasis in diabetes and obesity. Nat. Commun..

[42] Cote CD (2015). Resveratrol activates duodenal Sirt1 to reverse insulin resistance in rats through a neuronal network. Nat. Med.

[43] Duca FA (2015). Metformin activates a duodenal Ampk-dependent pathway to lower hepatic glucose production in rats. Nat. Med..

[44] Thaler JP (2012). Obesity is associated with hypothalamic injury in rodents and humans. J. Clin. Invest.

[45] Samuel VT (2009). Fasting hyperglycemia is not associated with increased expression of PEPCK or G6Pc in patients with type 2 diabetes. Proc. Natl. Acad. Sci. USA.

[46] Karamanlis A (2007). Effects of protein on glycemic and incretin responses and gastric emptying after oral glucose in healthy subjects. Am. J. Clin. Nutr..

[47] Duraffourd C (2012). Mu-opioid receptors and dietary protein stimulate a gut-brain neural circuitry limiting food intake. Cell.

[48] Duca FA, Bauer PV, Hamr SC, Lam TK (2015). Glucoregulatory relevance of small intestinal nutrient sensing in physiology, bariatric surgery, and pharmacology. Cell Metab..

[49] Rasmussen BA (2012). Duodenal activation of cAMP-dependent protein kinase induces vagal afferent firing and lowers glucose production in rats. Gastroenterology.

[50] Batterham RL (2006). Critical role for peptide YY in protein-mediated satiation and body-weight regulation. Cell Metab..

[51] Fei YJ (1994). Expression cloning of a mammalian proton-coupled oligopeptide transporter. Nature.

[52] Erickson RH, Gum JR, Lindstrom MM, McKean D, Kim YS (1995). Regional expression and dietary regulation of rat small intestinal peptide and amino acid transporter mRNAs. Biochem. Biophys. Res. Commun..

[53] Ihara T, Tsujikawa T, Fujiyama Y, Bamba T (2000). Regulation of PepT1 peptide transporter expression in the rat small intestine under malnourished conditions. Digestion.

[54] Thamotharan M, Bawani SZ, Zhou X, Adibi SA (1999). Functional and molecular expression of intestinal oligopeptide transporter (Pept-1) after a brief fast. Metabolism.

[55] Nassl AM (2011). Amino acid absorption and homeostasis in mice lacking the intestinal peptide transporter PEPT1. Am. J. Physiol. Gastrointest. Liver Physiol..

[56] Chu XY (2001). Correlation between epithelial cell permeability of cephalexin and expression of intestinal oligopeptide transporter. J. Pharmacol. Exp. Ther..

[57] Naruhashi K, Sai Y, Tamai I, Suzuki N, Tsuji A (2002). PepT1 mRNA expression is induced by starvation and its level correlates with absorptive transport of cefadroxil longitudinally in the rat intestine. Pharm. Res..

[58] Iakoubov R, Ahmed A, Lauffer LM, Bazinet RP, Brubaker PL (2011). Essential role for protein kinase Czeta in oleic acid-induced glucagon-like peptide-1 secretion in vivo in the rat. Endocrinology.

[59] Shen H, Smith DE, Brosius FC3rd (2001). Developmental expression of PEPT1 and PEPT2 in rat small intestine, colon, and kidney. Pediatr. Res..

[60] Pan X, Terada T, Okuda M, Inui K (2004). The diurnal rhythm of the intestinal transporters SGLT1 and PEPT1 is regulated by the feeding conditions in rats. J. Nutr..

[61] Thamotharan M, Bawani SZ, Zhou X, Adibi SA (1999). Hormonal regulation of oligopeptide transporter pept-1 in a human intestinal cell line. Am. J. Physiol..

[62] Buyse M (2001). PepT1-mediated epithelial transport of dipeptides and cephalexin is enhanced by luminal leptin in the small intestine. J. Clin. Invest..

[63] Ziegler TR (2002). Distribution of the H+/peptide transporter PepT1 in human intestine: up-regulated expression in the colonic mucosa of patients with short-bowel syndrome. Am. J. Clin. Nutr..

[64] Hindlet P (2009). Reduced intestinal absorption of dipeptides via PepT1 in mice with diet-induced obesity is associated with leptin receptor down-regulation. J. Biol. Chem..

[65] Richards P (2016). High fat diet impairs the function of glucagon-like peptide-1 producing L-cells. Peptides.

[66] Watanabe K, Terada K, Sato J (2003). Intestinal absorption of cephalexin in diabetes mellitus model rats. Eur. J. Pharm. Sci..

[67] Gangopadhyay A, Thamotharan M, Adibi SA (2002). Regulation of oligopeptide transporter (Pept-1) in experimental diabetes. Am. J. Physiol. Gastrointest. Liver Physiol..

[68] Buse JB (2016). The primary glucose-lowering effect of metformin resides in the gut, not the circulation: results from short-term pharmacokinetic and 12-week dose-ranging studies. Diabetes Care.

[69] Zadeh-Tahmasebi M (2016). Activation of short and long chain fatty acid sensing machinery in the ileum lowers glucose production in vivo. J. Biol. Chem..

[70] Matthews DM, Muir GG, Baron DN (1964). Estimation of alpha-amino nitrogen in plasma and urine by the colorimetric ninhydrin reaction. J. Clin. Pathol..

