# Supplementary material for: Physiological and therapeutic regulation of glucose homeostasis by upper small intestinal PepT1-mediated protein sensing
Source: Nat Commun. 2018 Mar 16;9:1118. doi: 10.1038/s41467-018-03490-8 (PMC5856761; doi:10.1038/s41467-018-03490-8)
Supplement: Supplementary file 1 — Supplementary Information(PDF 493 kb) [file 41467_2018_3490_MOESM1_ESM.pdf]

Physiological and therapeutic regulation of glucose homeostasis by upper small intestinal PepT1-mediated protein sensing (Dranse et al.)

Supplementary information

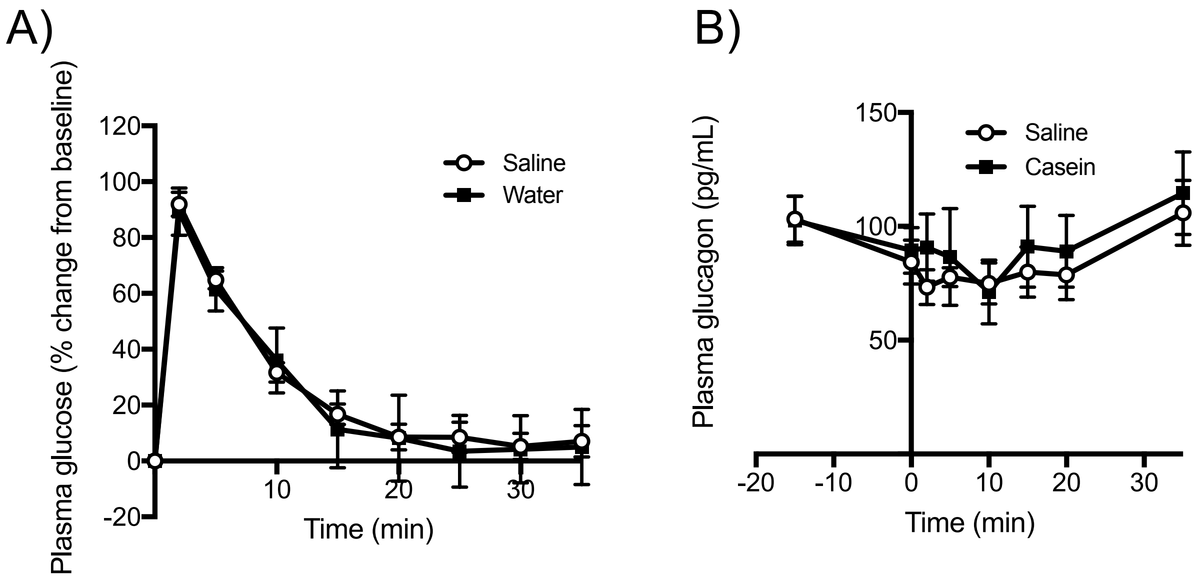

Supplementary Figure 1. *Extended data related to Figure 1.* Rats that received an upper small intestinal infusion of saline (n=12) or water (titrated similarly to the 8% casein solution, final pH=5.0, n=4) were subjected to an intravenous glucose tolerance test (IVGTT) as outlined in Fig. 1b (A). Plasma glucagon levels (B) over time during the IVGTT in rats that received an upper small intestinal (S.I.) infusion of saline (n=10) or 8% casein hydrolysate (n=8). Values are presented as mean ± s.e.m.where no significant difference was found between groups. Statistical significance at an individual timepoint was determined using an unpaired, two-tailed t-test.

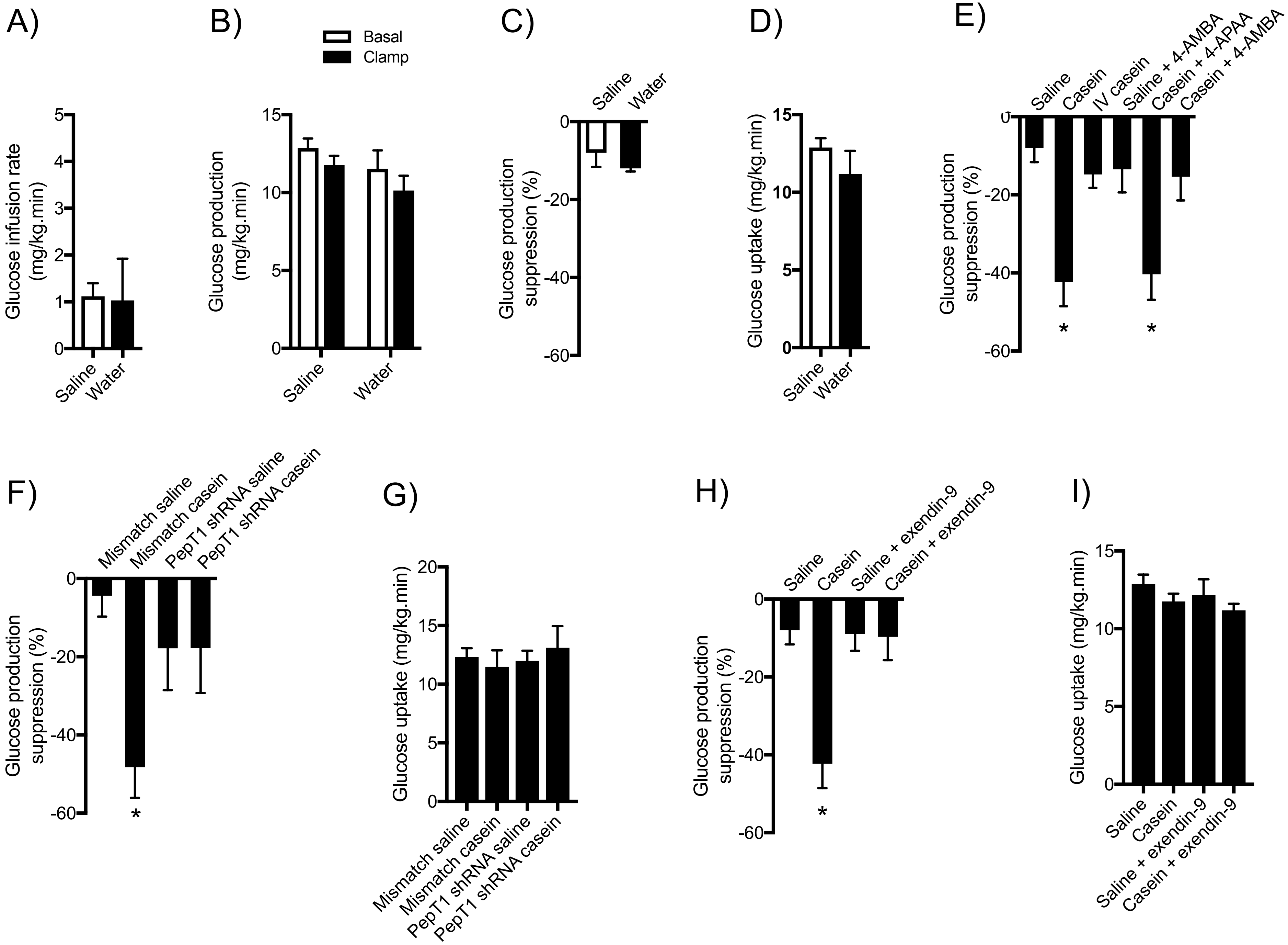

A)
Glucose infusion rate (mg/kg.min)
Saline
Water
B)
Basal
Clamp
Glucose production (mg/kg.min)
Saline
Water
C)
Saline
Water
Glucose production suppression (%)
D)
Glucose uptake (mg/kg.min)
Saline
Water
E)
Saline
Casein
IV casein
Saline + 4-AMBA
Casein + 4-APAA
Casein + 4-AMBA
Glucose production suppression (%)
*
*
F)
Mismatch saline
Mismatch casein
PepT1 shRNA saline
PepT1 shRNA casein
Glucose production suppression (%)
*
G)
Glucose uptake (mg/kg.min)
Mismatch saline
Mismatch casein
PepT1 shRNA saline
PepT1 shRNA casein
H)
Saline
Casein
Saline + exendin-9
Casein + exendin-9
Glucose production suppression (%)
*
I)
Glucose uptake (mg/kg.min)
Saline
Casein
Saline + exendin-9
Casein + exendin-9

Supplementary Figure 2. *Extended data related to Figure 3.* Rats that received an upper small intestinal (S.I.) infusion of saline (n=10) or water (titrated similarly to the 8% casein solution, final pH 5.0, n=3) were subjected to the basal insulin euglycemic pancreatic clamp as outlined in Fig. 3a. Rates of glucose infusion (A), glucose production (GP, B), percent GP suppression (C), and glucose uptake (D) were not significantly different between saline- and water- treated animals. Percent GP suppression for rats that received upper S.I. infusions of saline (n=10) or casein (n=9), intravenous (IV) casein (n=5), or upper S.I. infusions of saline + 4-AMBA (n=6), casein + 4-APAA (n=5), or casein + 4-AMBA (n=6) (E) during the pancreatic clamp. Percent GP suppression (F) and rate of glucose uptake (G) for rats that received mismatch shRNA lentiviral infection + saline infusion (n=6), mismatch + casein infusion (n=6), PepT1 shRNA lentiviral infection + saline infusion (n=5), or PepT1 shRNA + casein infusion (n=6) during the clamp. Percent GP suppression (H) and rate of glucose uptake (I) for rats that received saline + exendin-9 (n=3) or casein + exendin-9 (n=6) during the clamp. Values are presented as mean ± s.e.m., where basal represents the average GP of t=60-90, clamp represents the average GP of t=190-200, and * represents $p<0.05$ compared to saline control. Statistical significance was determined using an unpaired, two-tailed t-test (two groups) or ANOVA with a Tukey post-hoc test (3+ groups).

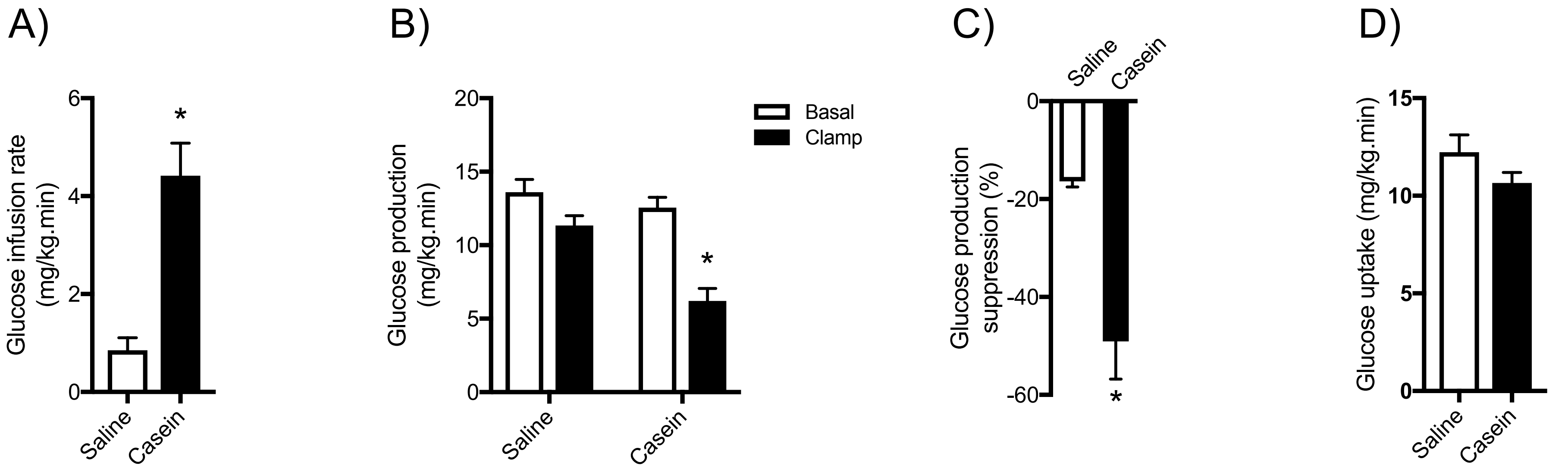

Supplementary Figure 3. *Casein decreases glucose production in rats that received an extended recovery period.* Rats were allowed to recover for 7-8 d following the surgical procedure and were subjected to the basal insulin euglycemic pancreatic clamp as outlined in Fig. 3a. Rates of glucose infusion (A), glucose production (GP, B), percent GP suppression (C), and glucose uptake (D) in rats that received an upper small intestinal infusion of saline (n=4) or casein (n=6). Values are presented as mean ± s.e.m., where basal represents the average GP of t=60-90, clamp represents the average GP of t=190-200, and * represents $p<0.05$ compared to saline control. Statistical significance was determined using an unpaired, two-tailed t-test (two groups) or ANOVA with a Tukey post-hoc test (3+ groups).

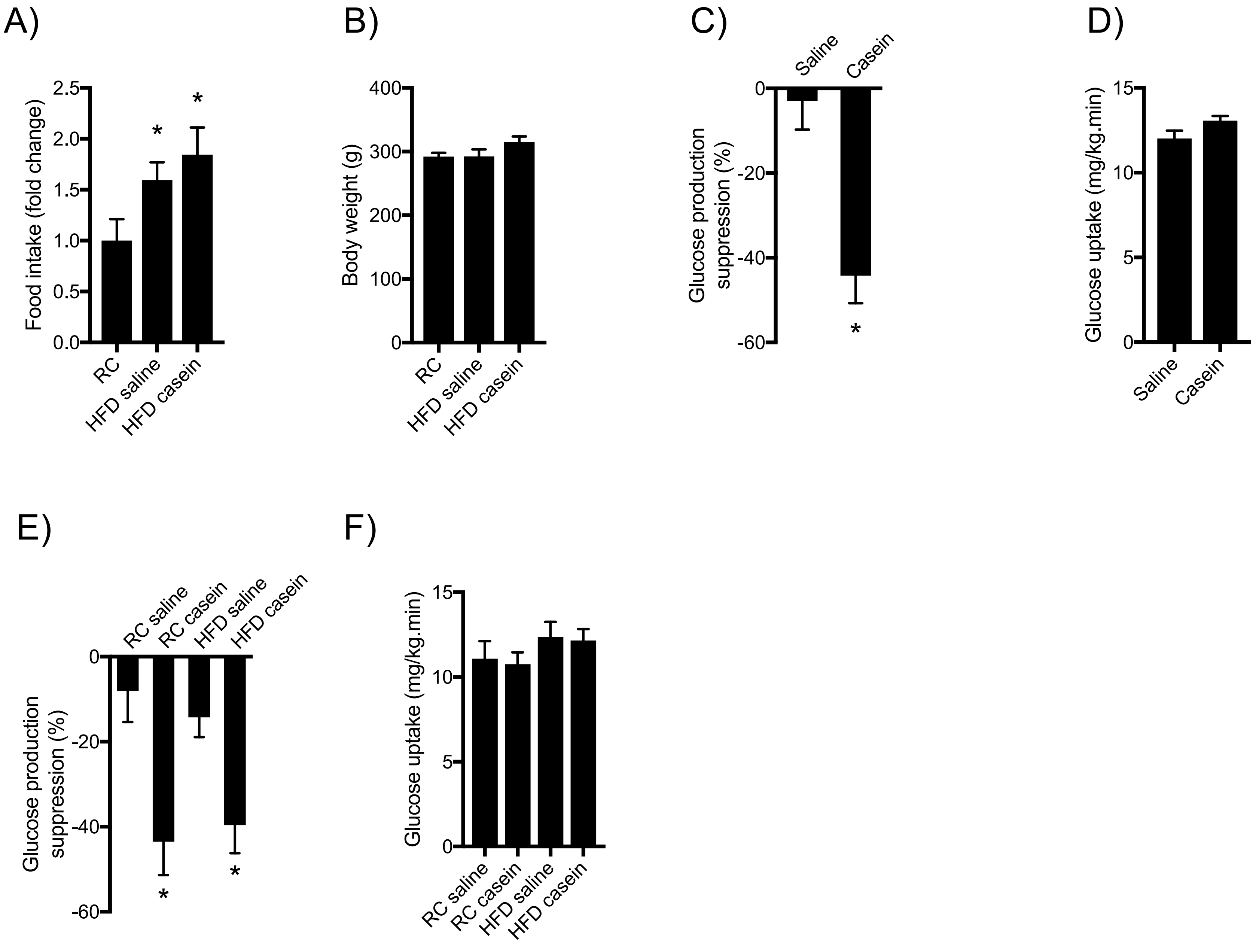
A)
Food intake (fold change)
2.5
2.0
1.5
1.0
0.5
0.0
*
*
RC
HFD saline
HFD casein
B)
Body weight (g)
400
300
200
100
0
RC
HFD saline
HFD casein
C)
Saline
Casein
Glucose production suppression (%)
0
-20
-40
-60
*
D)
Glucose uptake (mg/kg.min)
15
10
5
0
Saline
Casein
E)
RC saline
RC casein
HFD saline
HFD casein
Glucose production suppression (%)
0
-20
-40
-60
*
*
F)
Glucose uptake (mg/kg.min)
15
10
5
0
RC saline
RC casein
HFD saline
HFD casein

Supplementary Figure 4. *Extended data related to Figure 5.* Rats were fed a regular chow (RC) diet or a high-fat diet (HFD) (nutritional information available in Supplementary Table 1) for 3 d prior to performing the basal insulin euglycemic pancreatic clamp. Cumulative food intake (A) and body weight (B) were calculated on the morning of the pancreatic clamp (for all groups, n=6). Rates of percent glucose suppression (C) and glucose uptake (D) in 3 d HFD-fed rats that received an upper small intestinal infusion of saline (n=7) or casein (n=6). Rats were fed a RC or HFD for 28 d prior to performing the basal insulin euglycemic pancreatic clamp. Rates of percent glucose suppression (E) and glucose uptake (F) were monitored in rats that were fed either RC or HFD for 28 d and received an upper intestinal infusion of saline (n=5-6) or casein (n=5-8). Values are presented as mean ± s.e.m.where * represents $p<0.05$ compared to RC (panel A) or to respective saline control (panels C and G). Statistical significance was determined using an unpaired, two-tailed t-test (two groups) or ANOVA with a Tukey post-hoc test (3+ groups).

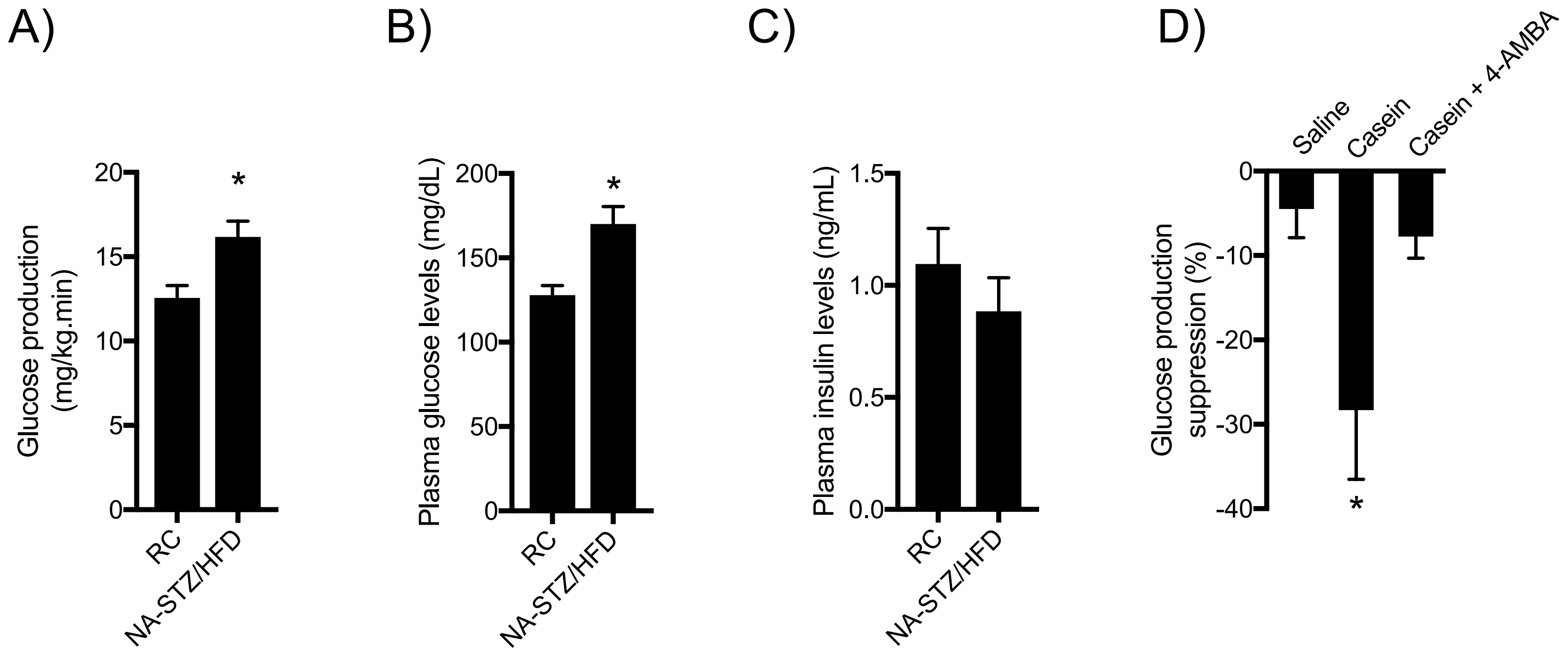

Supplementary Figure 5. *Extended data related to Figure 6.* Basal glucose production (A), plasma glucose levels (B), and plasma insulin levels (C) in healthy RC rats (n=6) or nicotinamide-streptozotocin/HFD-induced (NA-STZ/HFD) hyperglycemic rats (n=6). Percent glucose production suppression (D) in hyperglycemic rats that received an upper small intestinal infusion of saline (n=6), casein (n=7), or casein + 4-AMBA (n=6). Values are presented as mean ± s.e.m.where * represents $p<0.05$ compared to RC (panels A-B) or to saline control (panel D). Statistical significance was determined using an unpaired, two-tailed t-test (two groups) or ANOVA with a Tukey post-hoc test (3+ groups).

Supplementary Table 1. *Nutritional composition of diets used in the study*

Calories provided	Regular chow	Low protein	High protein	High fat
Carbohydrate (%)	49	65.3	21.3	44
Protein (%)	33	21.5	65.4	22
Fat (%)	18	13.1	13.4	34
Saturated	2.1	-	-	5.5
Monounsaturated	2.4	-	-	5.7
Polyunsaturated	1.2	-	-	1.8
Total calorie provided (kcal/g)	3.1	3.8	3.7	3.9

Supplementary Table 2. *Typical amino acid composition of casein hydrolysate (as provided by manufacturer)*

Amino acid	% (w/w)
Glutamic acid	16.5
Proline	8.5
Leucine	6.5
Lysine	6.4
Aspartic acid	5.5
Valine	5.3
Isoleucine	4.5
Serine	4.5
Phenylalanine	3.8
Threonine	3.6
Arginine	2.9
Alanine	2.4
Methionine	2.4
Histidine	2.1
Tyrosine	1.8
Glycine	1.6
Tryptophan	0.95
Cysteine	0.67

Supplementary Table 3. *Body weight, plasma glucose levels, and plasma insulin levels for groups receiving upper small intestinal infusion of saline or casein during the IVGTT (16 h fasted) and pancreatic clamp (4-6 h fasted).*

	IVGTT (Figure 1)		Clamp (Figure 3)	
	Saline n=18	Casein n=24	Saline n=10	Casein n=10
**Body weight**	277.6 ± 6.0	272.3 ± 3.1	292.2 ± 6.1	292.3 ± 8.2
**Basal**				
Glucose (mg/dL)	129.0 ± 3.0	130.7 ± 3.6	128.0 ± 5.6	131.5 ± 5.1
Insulin (ng/mL)	0.4 ± 0.1	0.5 ± 0.1	0.8 ± 0.2	0.9 ± 0.2
**Clamp**				
Glucose (mg/dL)	-	-	132.0 ± 6.8	122.3 ± 7.8
Insulin (ng/mL)	-	-	0.8 ± 0.2	0.9 ± 0.1

Values are presented as mean ± SEM where “basal” represents measurements at t=-15 min (IVGTT) or the average of t=60-90 min (pancreatic clamp), and “clamp” represents the average of t=190-200.
